# The Phospholipid Scramblases 1 and 4 Are Cellular Receptors for the Secretory Leukocyte Protease Inhibitor and Interact with CD4 at the Plasma Membrane

**DOI:** 10.1371/journal.pone.0005006

**Published:** 2009-03-31

**Authors:** Bénédicte Py, Stéphane Basmaciogullari, Jérôme Bouchet, Marion Zarka, Ivan C. Moura, Marc Benhamou, Renato C. Monteiro, Hakim Hocini, Ricardo Madrid, Serge Benichou

**Affiliations:** 1 Institut Cochin, Université Paris-Descartes, CNRS, UMR 8104, Paris, France; 2 INSERM, U567, Paris, France; 3 INSERM U699, Paris, France; 4 Université Paris 7-Denis Diderot, site Bichat, Paris, France; 5 INSERM U743, Paris, France; Comprehensive AIDS Reseach Center, China

## Abstract

Secretory leukocyte protease inhibitor (SLPI) is secreted by epithelial cells in all the mucosal fluids such as saliva, cervical mucus, as well in the seminal liquid. At the physiological concentrations found in saliva, SLPI has a specific antiviral activity against HIV-1 that is related to the perturbation of the virus entry process at a stage posterior to the interaction of the viral surface glycoprotein with the CD4 receptor. Here, we confirm that recombinant SLPI is able to inhibit HIV-1 infection of primary T lymphocytes, and show that SLPI can also inhibit the transfer of HIV-1 virions from primary monocyte-derived dendritic cells to autologous T lymphocytes. At the molecular level, we show that SLPI is a ligand for the phospholipid scramblase 1 (PLSCR1) and PLSCR4, membrane proteins that are involved in the regulation of the movements of phospholipids between the inner and outer leaflets of the plasma membrane. Interestingly, we reveal that PLSCR1 and PLSCR4 also interact directly with the CD4 receptor at the cell surface of T lymphocytes. We find that the same region of the cytoplasmic domain of PLSCR1 is involved in the binding to CD4 and SLPI. Since SLPI was able to disrupt the association between PLSCR1 and CD4, our data suggest that SLPI inhibits HIV-1 infection by modulating the interaction of the CD4 receptor with PLSCRs. These interactions may constitute new targets for antiviral intervention.

## Introduction

Secretory leukocyte protease inhibitor (SLPI) is a polypeptide of 132 residues (11.7 kDa) secreted by epithelial cells in all the mucous liquids such as saliva, bronchial and nasal secretions, cervical mucus, as well as in the seminal liquid [1]. SLPI is a powerful serine-protease inhibitor, and its main biological role is to ensure protection of tissues from degradation by the leukocyte proteolytic enzymes produced during local inflammatory reactions [2]. Found in the extracellular medium, SLPI is able to cross the biological membranes and to penetrate into the cell where it will exert some of its biological functions [3], [4]. At the structural level, the primary amino acid (a.a.) sequence of SLPI reveals the presence of two so-called «whey-acidic-protein» (WAP) motifs, a domain of about fifty a.a. with eight highly conserved cysteine residues that form four disulphide bonds. WAP motifs are specifically found in a family of inhibitors of serine-proteases, such as elastase, trypsin and chymotrypsin, whith SLPI and elafin (or trappin-2) being the most characterized members (for review, [5]) displaying both anti-inflammatory and antimicrobial activities.

Interestingly, several groups have shown that SLPI also displays, at physiological concentrations found in saliva (20–150 µg/ml) [6], [7], a specific antiviral activity against human immunodeficiency virus (HIV-1) [7]–[13]. The high salivary concentrations of SLPI may be responsible for the absence of oral transmission of HIV-1 [14]–[16], and for the reduced mother-to-child HIV-1 transmission by the mother's milk [6]. Similarly, high concentrations of SLPI in vaginal fluids have been associated with reduced rates of perinatal HIV-1 transmission [17]. The inhibition of HIV-1 replication by SLPI is independent of its anti-protease activity, but is related to a perturbation of the virus entry process at a stage posterior to the interaction of the viral surface glycoprotein gp120 with the CD4 receptor at the surface of HIV-1 target cells [9], [11]. The SLPI antiviral activity is indeed observed in various cell culture models, including CD4-positive lymphoid and monocytoid cell lines as well as primary lymphocytes and monocyte-derived macrophages. It is exerted, independently of the chemokine coreceptor usage, on viral strains with tropism for either lymphocytes (×4 strains) or macrophages (R5 strains) [7], [8], [13].

SLPI appears to block HIV-1 entry by interacting with a non-CD4 cell membrane receptor protein [9], [11]. Only two membrane-associated proteins able to interact with SLPI have been identified so far, the phospholipid (PL) binding protein annexin II and the phospholipid scramblase 1 (PLSCR1) [9], [18]. While annexin II was proposed as a cofactor specifically involved in the SLPI antiviral activity observed on macrophages [9], this finding does not explain the ability of SLPI to block HIV-1 entry both in CD4-positive transformed T-cell lines and primary peripheral blood lymphocytes [7], [8], [10], [13], [19] where annexin II does not seem to be expressed [11], [20].

Therefore, we further explored the specific role of the interaction of SLPI with the host cell PLSCR1 protein in the anti-HIV-1 inhibitory activity of SLPI. PLSCR1 is a membrane protein of 318 amino acids (a.a.) that is widely expressed at the surface of many cell types [21]–[23]. It is one of the four members of the scramblase family identified in humans, which share between 46 and 59% a.a. identity in their primary sequences [21]. Whereas PLSCR1, 3 (295 a.a.) and 4 (329 a.a.) are widely expressed in most human tissues, expression of PLSCR2 (224 a.a.) is restricted to testis. Like other human and murine scramblases, PLSCR1 shows a type-II membrane organization, and comprises a long cytoplasmic domain of 290 a.a., followed by a single transmembrane helix (a.a. 291–309) and a short C-terminal extracellular domain corresponding to the last 9 a.a. [22], [23]. Scramblases are membrane proteins allowing the bidirectional and nonspecific transbilayer movement of phospholipids across the plasma membrane ([22], [24], [25], for reviews).

Here, we confirm that recombinant SLPI is able to inhibit HIV-1 infection of primary T lymphocytes when used at the physiological concentrations found in saliva, and we show that SLPI can also inhibit the transfer of HIV-1 virions from dendritic cells derived from primary monocytes towards autologous T lymphocytes, a process which is important in vivo for virus spreading after mucosal transmission (for review, [26]). At the molecular level, we show that SLPI is a ligand for both PLSCR1 and PLSCR4, and we reveal that PLSCR1 and PLSCR4 interact directly with the CD4 receptor at the cell surface of T lymphocytes. Since the same region of the cytoplasmic domain of PLSCR1 is involved in the binding with CD4 and SLPI, our data suggest a model in which the inhibitory effect of SLPI results from its ability to modulate the interaction of the CD4 receptor with scramblases. The disruption of these interactions between CD4 and scramblases may thus represent new targets for antiviral intervention.

## Results

### Anti-HIV-1 activity of recombinant SLPI

**Figure 1:**
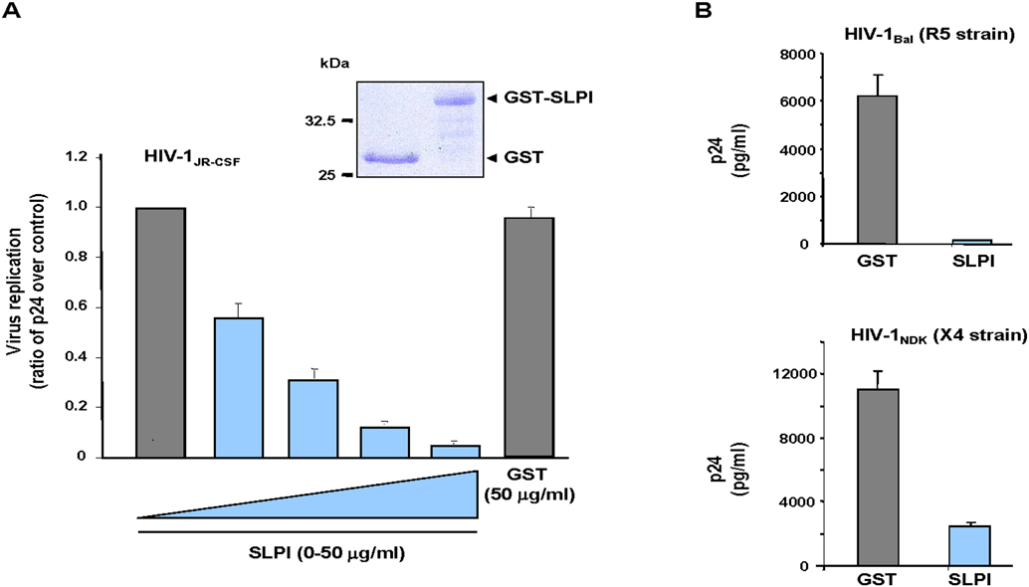
Anti-HIV-1 activity of recombinant SLPI. A) Inhibition of virus replication. Primary T lymphocytes were isolated from peripheral blood mononuclear cells and used for infection with the HIV-1JR-CSF isolate in the presence of increasing concentrations (0–50 µg/ml) of purified GST-SLPI (see inset, coomassie blue) or GST (50 µg/ml). After washing, infected cells were cultured for 6 days and the viral production was quantified by measuring the p24 production in the cell-culture medium. B) Inhibition of virus transfer from dendritic cells to T lymphocytes. Dentritic cells were derived from purified primary monocytes, and incubated with either HIV-1Bal (R5 strain, upper panel) or HIV-1NDK (×4 strain, lower panel) for 1 h at 37°C in the presence of 50 µg/ml of purified GST-SLPI or GST. After washing, IL-2 activated-lymphocytes were added in a 1/5 ratio and maintained in co-culture for 72 h. Viral production was quantified by measuring the p24 production in the cell culture medium.

In order to further explore its antiviral activity against HIV-1, a recombinant form of SLPI, deleted of the 25 a.a. long signal peptide, and fused to GST (GST-SLPI) was expressed in *E. coli* and purified on GSH-agarose beads. As shown on Fig. 1A, GST-SLPI was able to inhibit, in a dose-dependent manner, the replication of a primary viral strain (HIV-1JR-CSF) in human primary T lymphocytes isolated from peripheral blood. The highest activity was obtained at a concentration of 50 µg/ml of GST-SLPI, which corresponds to the physiological concentrations usually found in saliva or in endocervical and seminal fluids [6], [7]. By contrast, no significant inhibition was observed at the same concentration of the GST control protein. In addition, GST-SLPI inhibited the transfer towards autologous T lymphocytes of HIV-1 virions from dendritic cells derived from primary blood monocytes (Fig. 1B). Interestingly, this inhibitory activity was exerted on viral strains with tropism for either lymphocytes (HIV-1NDK ×4 strain) or macrophages (HIV-1Bal R5 strain). These results show that recombinant SLPI inhibits replication of HIV-1 in primary T lymphocytes, but also cell-to-cell transfer of the virus from dendritic cells to T lymphocytes.

### SLPI directly binds to the phospholipid scramblases 1 and 4

**Figure 2:**
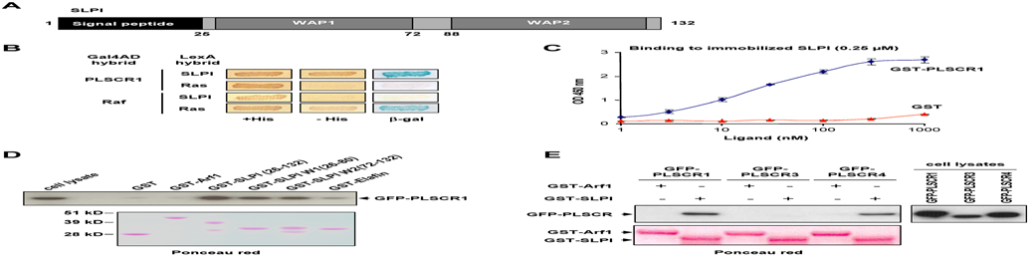
Characterization of the PLSCR1/SLPI interaction. A) Schematic representation of SLPI. SLPI contains an N-terminal signal peptide (black) and two WAP motifs (grey); a.a. are numbered according to Tseng et al. [18]. B) Interaction in the two-hybrid system. L40 yeast strain expressing the indicated Gal4AD and LexA hybrids was analyzed for histidine auxotrophy and β-gal activity. Transformants were patched on medium with histidine (left panels) and then replica-plated on medium without histidine (central panels) and on Whatman filter for β-gal assay (right panels). Growth in the absence of histidine and expression of β-gal activity indicate the interaction between hybrids. C) ELISA interaction analysis. 96-well plates were coated with non-tagged SLPI at a final concentration of 0.25 µM. After washings, GST-PLSCR1 (black curve) or GST (red curves) was incubated at concentrations ranging from 1 nM to 1 µM. Binding was then revealed with an anti-GST antibody and a secondary peroxidase-conjugated anti-mouse IgG. The peroxidase substrate solution was incubated for 10 min and the optical density was measured at 450 nm. D and E) In vitro interactions. Lysates from 293T cells expressing GFP-PLSCR1, GFP-PLSCR3 or GFP-PLSCR4 were incubated with equal amounts of GST fusions (lower panels, Ponceau red) immobilized on GSH-sepharose beads as indicated at the top. Bound proteins were then analyzed by immunoblotting with anti-GFP (upper panels).

Since it has been previously suggested that the ubiquitous PLSCR1 membrane protein was a receptor for SLPI [18], we further explored this possibility by using several interaction assays. The interaction between SLPI and PLSCR1 was initially analyzed in the yeast two-hybrid system. The SLPI sequence was fused to LexA and assessed for interaction with PLSCR1 fused to the Gal4AD in the L40 yeast strain containing the two LexA-inducible reporter genes, *HIS3* and *LacZ*. In contrast to the results published previously [18], the full length 132 a.a. long form of SLPI, containing the N-terminal signal peptide (see on Fig. 2A), was not able to interact with PLSCR1 in this assay (data not shown), whereas a specific interaction could be detected with a form of SLPI lacking the first 25 residues, as evidenced by growth of yeast cells on medium without histidine and expression of the β-gal activity (Fig. 2B). This form of SLPI (a.a. 26–132) corresponds to the mature secreted protein after cleavage of the N-terminal signal sequence. Binding of SLPI to PLSCR1 was direct, since we were able to recapitulate *in vitro* this interaction in an ELISA test where immobilized SLPI specifically captured the purified GST-PLSCR1 fusion protein but not the GST control (Fig. 2C).

The specificity of the interaction was also confirmed by biochemical approaches. GST-SLPI(26-132) was first immobilized on GSH-sepharose beads, and then incubated with a lysate from cells expressing GFP-PLSCR1. Bound proteins were analyzed by Western blotting with anti-GFP (Fig. 2D). GFP-PLSCR1 specifically bound to GST-SLPI, but not to GST or to GST-Arf1 used as negative controls. As shown in Fig. 2D, the two WAP1 (GST-SLPI W1) and WAP2 (GST-SLPI W2) motifs of SLPI were separately able to recruit GFP-PLSCR1 from tranfected cells, but to a lower extent compared with the mature form of SLPI(26-132), suggesting that both motifs cooperate to allow an optimal interaction with PLSCR1. A weak interaction was also detected in the same pull-down assay with a recombinant form of the mature elafin (GST-Elafin) (Fig. 2D), another member of the family of serine protease inhibitors containing a single WAP motif [5].

Similarly, we analyzed whether SLPI was also able to interact with PLSCR3 and PLSCR4, the two other human scramblases that are widely expressed in all tissues. As shown in Fig. 2E, both GFP-PLSCR1 and GFP-PLSCR4, but not GFP-PLSCR3, were specifically pulled-down on GST-SLPI-immobilized beads. These results indicate that SLPI is a specific ligand for both PLSCR1 and PLSCR4.

### Co-distribution of scramblases and CD4 at the plasma membrane

**Figure 3:**
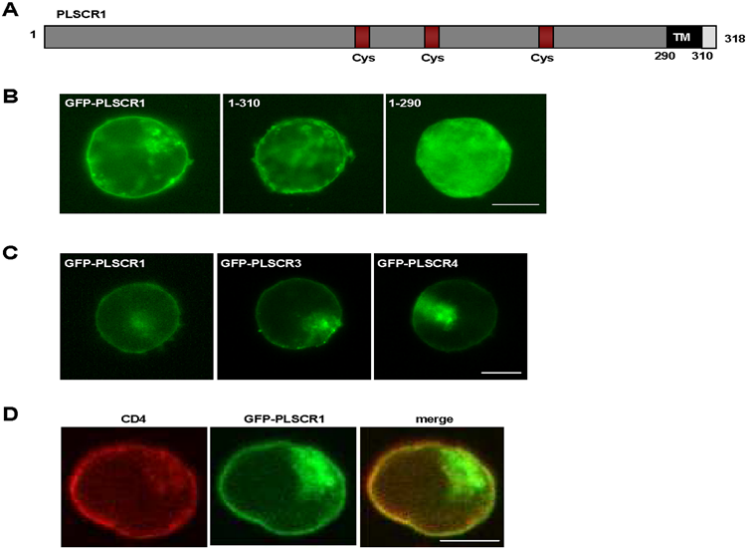
Co-distribution of scramblases and CD4 at the plasma membrane. A) Schematic representation of PLSCR1. PLSCR1 is a type-II transmembrane protein with a long N-terminal cytoplasmic domain (a.a. 1–290, grey box) containing 3 Cys-rich motifs (red boxes), and a transmembrane domain (a.a. 291–310, black box). Amino acids are numbered according to Zhou et al. [23]. B) Localization of wild type and deleted GFP-PLSCR1 forms. Jurkat CD4-positive T cells expressing the full length (left panel) or deleted forms (1–310 and 1–290, central and right panels, respectively) of GFP-PLSCR1 were fixed and directly examined. Cells were analyzed by epifluorescence microscopy, and images were acquired using a CCD camera. C) Localization of GFP-PLSCR3 and GFP-PLSCR4. Jurkat cells expressing GFP-PLSCR1 (left panel), GFP-PLSCR3 (central panel) or GFP-PLSCR4 (right panel) were fixed and directly examined as in (B). D) Subcellular distribution of CD4 and PLSCR1. Jurkat cells expressing wild type GFP-PLSCR1 (middle panel) were fixed, permeabilized and subsequently stained with an anti-CD4 (left panel). Scale bars, 10 µm.

Molecular modeling predicted that PLSCR1 is a type II membrane protein with a single transmembrane helix and a short C-terminal extracellular region (see on Fig. 3A). To determine whether PLSCR1 was expressed at the plasma membrane in CD4-positive T cells, we first analyzed the cellular distribution of the wild type or truncated forms of PLSCR1 fused to the C-terminus of GFP in Jurkat T cells. After transfection, the cells were fixed and directly examined by fluorescence microscopy (Fig. 3B). As expected, both the full length GFP-PLSCR1 fusion and the (1–310) truncated form, lacking the last 8 C-terminal a.a., were predominantly localized as a rim staining at the plasma membrane (left and central panels, respectively), but a consistent fraction was also observed in an intracellular membrane compartment which largely co-localized with Golgi markers (not shown). By contrast, the truncated form (1–290) deleted of the last 28 C-terminal a.a. including the transmembrane domain showed a diffuse nucleo-cytoplasmic distribution (right panel). Therefore, PLSCR1, as well as PLSCR3 and PLSCR4 (Fig. 3C), are transmembrane proteins which are largely localized at the plasma membrane in CD4-positive T lymphoid cells. As expected, GFP-PLSCR1 and the endogenous CD4 antigen were both distributed at the plasma membrane in T cells (Fig. 3D).

### PLSCR1 and PLSCR4 interact directly with the CD4 receptor

**Figure 4:**
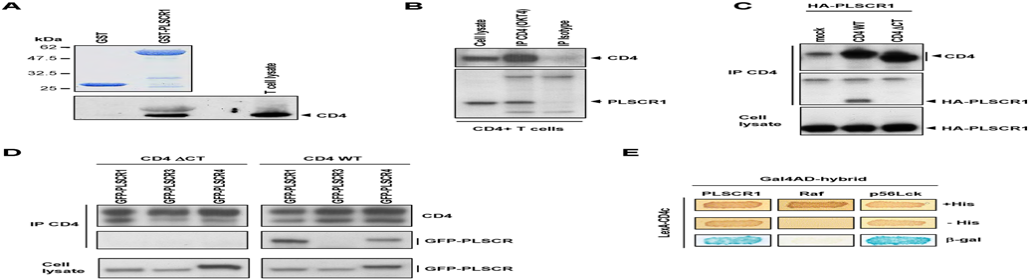
Interaction of PLSCR1 and PLSCR4 with the CD4 receptor. A) In vitro interaction. Lysates from Jurkat CD4-positive T cells were incubated with equal amounts of GST or GST-PLSCR1 (upper panel, coomassie blue) immobilized on GSH-sepharose beads. Bound proteins were then analyzed by immunoblotting with anti-CD4 (lower panel). B) Co-immunoprecipitation of endogenous proteins from T lymphocytes. Jurkat T cells were lyzed and CD4 was precipitated with either anti-CD4 (OKT4) or a control isotypic antibody. Precipitates were analyzed by Western blot with anti-CD4 (upper panel) or anti-PLSCR1 (lower panel). C) Co-precipitation of overexpressed CD4 and PLSCR1 proteins. 293T cells expressing HA-tagged PLSCR1 (lower panel, Cell lysate) in combination with wild-type CD4 (WT) or a mutant of CD4 deleted of its cytoplasmic domain (ΔCT) were lyzed and the CD4 forms were precipitated with anti-CD4. Precipitates were then analyzed by Western blot with anti-CD4 (upper panel) or anti-HA (middle panel). D) Co-precipitation of overexpressed CD4 and PLSCR4 proteins. 293T cells expressing GFP- PLSCR1, GFP-PLSCR3 or GFP-PLSCR4 (lower panels, Cell lysate) in combination with CD4 WT (right panels) or CD4 ΔCT (left panels) were lyzed and the CD4 forms were precipitated with anti-CD4. Precipitates were then analyzed as in (C). Of note, the differences in migration observed between PLSCR1 (318 a.a.), PLSCR3 (295 a.a.) and PLSCR4 (329 a.a.) is likely related to the respective amino acid lengths of the GFP fusion proteins [23]. E) Interactions in the two-hybrid system. L40 yeast strain expressing the LexA-CD4c hybrid in combination with the indicated Gal4AD hybrids was analyzed for histidine auxotrophy and β-gal activity. Transformants were patched on medium with histidine (upper panels) and then replica-plated on medium without histidine (middle panels) and on Whatman filter for β-gal assay (lower panels).

Since it was previously reported that the inhibition of HIV-1 replication by SLPI resulted from a direct action on the virus entry process [9], [11], we asked whether PLSCR1 could directly associate with CD4, the main receptor of HIV-1 at the cell surface of T lymphocytes. This hypothesis was first challenged in vitro by pull-down assay. Recombinant PLSCR1 fused to GST was expressed in *E. coli*, immobilized on GSH-sepharose beads and then tested for its ability to retain full length CD4 from a lysate of Jurkat T-cells. Bound proteins were analyzed by Western blot with anti-CD4 antibody (Fig. 4A). Endogenous CD4 was specifically pulled-down by GST-PLSCR1, but not by the GST control. Similarly, GST-PLSCR1 was also able to retain in vitro translated ^35^S-CD4 (data not shown), indicating a direct interaction between the two cellular proteins.

**Figure 5:**
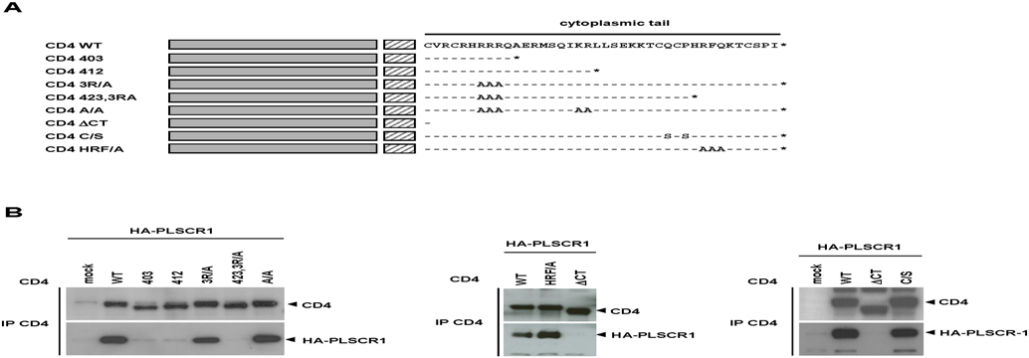
Mapping of the CD4 determinants required for PLSCR1 binding by co-immunoprecipitation assay. A) Schematic representation of the human CD4 mutants. The extracellular and transmembrane (TM) domains of CD4 are represented in grey and hatched, respectively. The a.a. sequences of the cytoplasmic tail of the CD4 mutants are aligned with that of the wild-type CD4 (CD4 WT). Dashes (−) indicate a.a. identities with the wild type protein and a.a. substitutions are identified. B) Co-immunoprecipitation assay. Protein extracts were prepared 48 h post transfection from 293T cells co-expressing HA-PLSCR1 together with wild type or mutated CD4 as indicated at the top. Crude extracts were then subjected to CD4 immunoprecipitation followed by Western blot analysis with anti-CD4 (upper panels) and anti-HA (lower panels).

The interaction between endogenous PLSCR1 and CD4 proteins was finally documented by a co-immunoprecipitation assay performed on CD4-positive T-lymphocytes. Jurkat cells were lyzed and CD4 was precipitated with an anti-CD4 antibody. Precipitates were then analyzed by Western blotting with anti-CD4 (Fig. 4B, upper panel) and anti-PLSCR1 (lower panel). Endogenous PLSCR1 was detected as a 35-kDa protein only from cell lysate immunoprecipitated with anti-CD4 and not from lysate treated with the control isotypic antibody, demonstrating that PLSCR1 interacts with CD4 in human CD4-positive T-lymphocytes. Interestingly, co-immunoprecipitation experiments performed on 293T cells expressing HA-tagged PLSCR1 in combination with wild type CD4 (WT) or a CD4 mutant lacking the cytoplasmic domain (ΔCT) revealed that the CD4 mutant failed to associate with PLSCR1 (Fig. 4C). In addition, similar experiments performed in 293T cells showed that PLSCR4 was also able to interact with CD4, and this interaction was again dependant of the cytoplasmic domain of the receptor (Fig. 4D). Since the results reported in Figs 4C and 4D indicated that the C-terminal cytoplasmic tail of CD4 (CD4c) was absolutely required to maintain the interactions, we analyzed whether this CD4c domain could be sufficient to associate with PLSCR1 in the two-hybrid system. The 40 a.a. long cytoplasmic domain of CD4 (see on Fig. 5A) was thus expressed in the L40 yeast reporter strain as a protein fused with LexA and in combination with the full length PLSCR1 fused to Gal4AD. As shown in Fig. 4E, the LexA-CD4c hybrid conferred on the reporter strain the ability to grow on medium without histidine and to express β-gal activity in the presence of the Gal4AD-PLSCR1 hybrid, but not in cells co-expressing an irrelevant Gal4AD-Raf hybrid. Used as control, we detected the well-characterized interaction between CD4c and the p56Lck kinase in this system (Fig. 4E).

Altogether, these results indicate that both PLSCR1 and PLSCR4 can interact with the CD4 receptor, and these interactions are mediated by the cytoplasmic domain of the CD4 receptor.

### Determinants of the CD4 cytoplasmic domain required for binding to PLSCR1

In order to characterize the determinants of the CD4c domain that participated in the interaction, a set of CD4 mutants with deletions and/or point mutations within the cytoplasmic domain (see Fig. 5A) were assessed for PLSCR1 binding by co-immunoprecipitation assay. CD4 mutants were expressed in 293T cells in combination with HA-PLSCR1, and then immunoprecitated with anti-CD4. As deduced from the experiments shown in Fig. 5B, the determinants required for PLSCR1 binding were localized in the distal portion of the cytoplasmic domain, since the CD4 423,3R/A mutant, lacking the last 10 a.a., failed to interact with PLSCR1 (left panel). However, mutations of the HRF residues found in position 425 to 427 (mutant CD4 HRF/A) did not affect binding to PLSCR1, suggesting that the last 6 residues are essential to maintain the interaction. We could note that the two cysteine residues, that are critical for binding to p56Lck ([27], and data not shown), are not required for binding to PLSCR1 (right panel, mutant CD4 C/S), indicating that the determinants required for interaction with PLSCR1 are different from those involved in the interaction with p56Lck.

**Figure 6:**
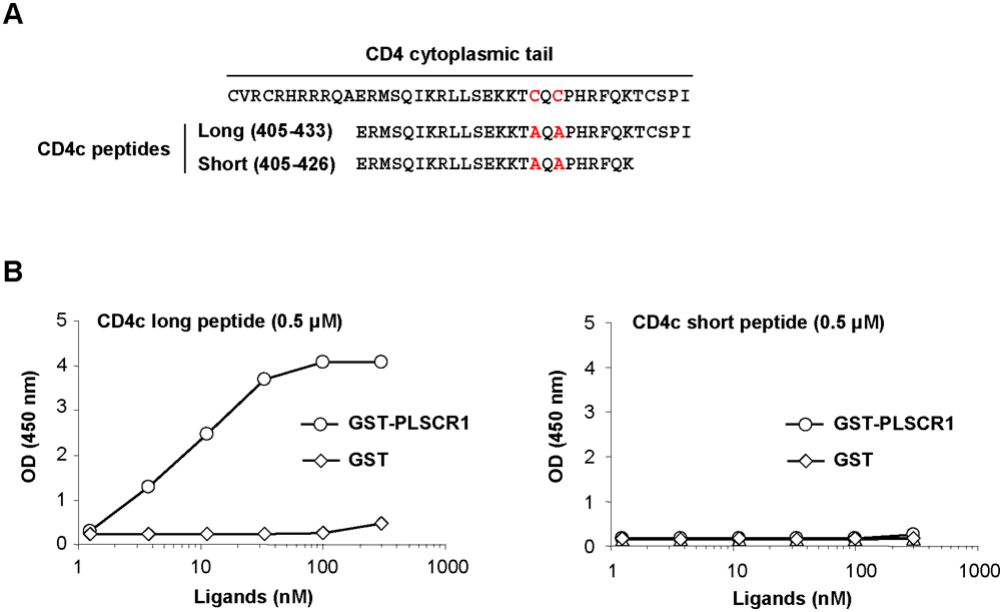
Mapping of the CD4 determinants required for PLSCR1 binding by ELISA. A) Primary a.a. sequences of the CD4 long (405–433) and short (405–426) peptides used in the ELISA test. The primary sequence of the entire cytoplasmic domain of CD4 is shown at the top; the 2 Cys residues required for p56Lck binding and mutated in Ala in both peptides are indicated in red. B) ELISA interaction test. 96-well plates were coated with the CD4c long (left panel) or short (right panel) peptides at a final concentration of 0.5 µM. After washings and blocking of non-specific binding sites, GST or GST-PLSCR1 was incubated at concentrations ranging from 1 nM to 300 nM. Binding was then revealed with an anti-GST antibody and a secondary peroxidase-conjugated anti-mouse IgG. The peroxidase substrate solution was incubated for 10 min and the optical density was measured at 450 nm.

To confirm that the distal region of CD4c was important to support PLSCR1 binding, we developed an ELISA to recapitulate the CD4-PLSCR1 interaction *in vitro*. Two peptides spanning the regions 405–433 and 405–426 of CD4 (Fig. 6A) were synthesized and then coated on microtiter plates. After incubation with purified GST-PLSCR1 or GST, binding was revealed with an anti-GST antibody. As shown in Fig. 6B, a positive signal was observed in a large range of GST-PLSCR1 concentrations with the long CD4c peptide (left panel), but not with the short CD4c peptide lacking the last 5 a.a. (right panel). This signal was specific, since no binding to the CD4c peptide was detected with the GST-Arf1 (not shown) and GST controls at concentrations ranging from 1 to 300 nM. Again, the cysteine residues directly involved in p56Lck binding were not required for PLSCR1 binding, since the CD4c peptides had alanine at these positions (Fig. 6A). These data definitively demonstrate that the interaction between PLSCR1 and CD4 is direct, and requires the last 5 residues of the CD4 cytoplasmic domain.

### SLPI binds to the cytoplasmic domain of PLSCR1 and impairs CD4/PLSCR1 association

**Figure 7:**
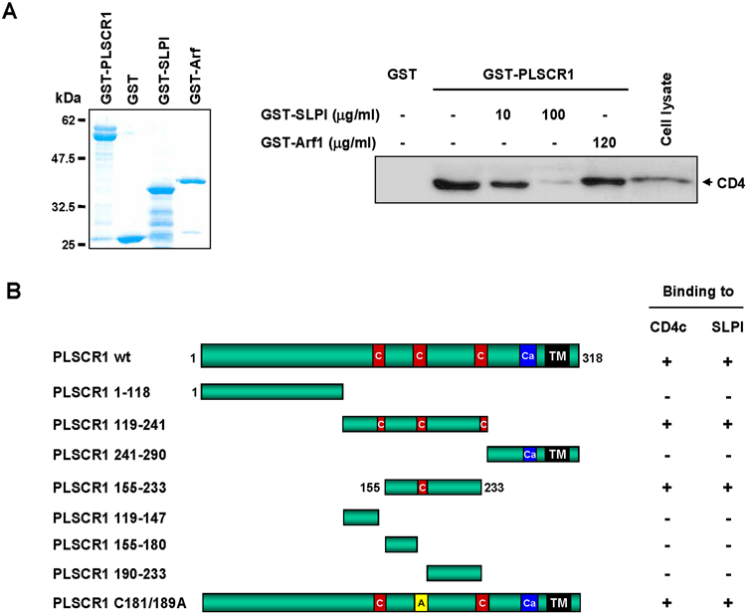
Disruption of the CD4/PLSCR1 interaction by SLPI. A) In vitro inhibition of the CD4/PLSCR1 interaction by SLPI. GST or GST-PLSCR1 (left panel, coomassie blue) was incubated with equal amounts of lysates from Jurkat CD4-positive T cells in the presence of the indicated concentrations of either GST-SLPI or GST-ARF1 (left panel) used as a control. Bound proteins were analyzed by Western blot with anti-CD4 (right panel). B) Mapping of the PLSCR1 determinants required for binding to CD4 and SLPI. L40 yeast strain expressing either the cytoplasmic domain of CD4 (CD4c) or SLPI fused to LexA in combination with each of the deleted forms of the Gal4AD-PLSCR1 hybrids indicated on the left was analyzed for histidine auxotrophy and beta-gal activity. The interactions between hybrid proteins were scored as follows: (+), cell growth on medium without histidine and development of a β-gal activity; (−), no growth on medium without histidine and no β-gal activity.

Since our results demonstrated that both CD4 and SLPI interacted with PLSCR1, we first investigated if the binding of SLPI to PLSCR1 could have any effect on CD4/PLSCR1 interaction. The impact of SLPI on the recruitment of CD4 by recombinant GST-PLSCR1 was analyzed using the pull-down assay described previously (Fig. 7A). At a concentration of 100 µg/ml, recombinant GST-SLPI (left panel) dramatically reduced the interaction of GST-PLSCR1 with native CD4 from a Jurkat cell lysate (right panel). By contrast, no significant inhibition of CD4 binding was observed with the GST-Arf1 (right panel) and GST (not shown) controls at the concentration of 120 µg/ml.

To determine the molecular nature of such an inhibition, we mapped the region of PLSCR1 involved in the interaction with the mature form of SLPI and with CD4c as well. Deletion mutants of PLSCR1 were generated and analyzed in the two-hybrid system for their capacity to interact with either the cytoplasmic domain of CD4 or the mature form (26–132) of SLPI. The results recapitulated in Fig. 7B show that CD4 and SLPI bound to the same region of PLSCR1 located in the cytoplasmic domain of the protein. The minimal sequence of PLSCR1 able to interact with both partners was mapped between residues 155 and 233 in a region containing the Cys-rich motif required for palmitoylation of the protein [28]. However, this motif did not seem to participate in these interactions, since Ala substitution of the 5 Cys residues (mutant PLSCR1 C181/189A) did not disturb the capacity of PLSCR1 to interact with either CD4 or SLPI. Interestingly, these results show that the interaction between CD4 and PLSCR1 is mediated by the respective cytoplasmic domains of these two membrane proteins.

Altogether, the results reported in Fig. 7 strongly suggest that SLPI modulates, at physiological concentrations (20–150 µg/ml) [6], [7], the CD4-PLSCR1 interaction by competitive binding to the CD4-binding region of PLSCR1.

## Discussion

In the present study, we document and highlight that the innate host defense SLPI polypeptide, secreted in all mucosal liquids and displaying an inhibitory activity against HIV-1, is a natural ligand for the ubiquitously expressed plasma membrane PLSCR1 protein. Initially revealed from a genetic two-hybrid screening performed to identify SLPI binding proteins [18], we confirm the specificity of the interaction with PLSCR1 by biochemical approaches, and reveal that SLPI also binds to PLSCR4, another member of the scramblase family. Since PLSCR1 is a type II integral membrane protein, we show that SLPI directly binds to a region of PLSCR1 located in the central part of its long cytoplasmic domain. Moreover, we demonstrate that PLSCR1, and also PLSCR4, can interact directly with CD4, the main receptor required for HIV-1 entry into its target cells, such as T lymphocytes and macrophages. These interactions are mediated by determinants found in the cytoplasmic domains of these integral membrane proteins. Interestingly, we demonstrate that CD4 and SLPI bind to the same region of the cytoplasmic domain of PLSCR1 (a.a. 155–233). Finally, we show that SLPI competes with CD4 thus modulating its interaction with PLSCR1.

While recent works focused on the biological functions of PLSCR1, nothing is known regarding the subcellular localization and the functions of PLSCR4 ([29], for review). PLSCR1 was originally identified as a membrane protein that promoted acceleration of transbilayer phospholipid movements in response to intracellular calcium, but increasing evidence indicates that PLSCR1 also has biological roles in cell signaling pathways. PLSCR1 has been shown to be engaged when cells are activated through several plasma membrane receptors, including the epidermal growth factor receptor and the high-affinity IgE receptor [30]. The long cytoplasmic domain of PLSCR1 contains multiple proline-rich motifs within its N-terminal distal region, and these motifs may constitute Src-homology 3 (SH3) domain binding sites, as revealed by the interaction of PLSCR1 with the SH3 domain of the c-Abl tyrosine kinase that in turn phosphorylates PLSCR1 [31]. However, the determinants of PLSCR1 required for SLPI binding are located in the central region of the cytoplasmic tail containing cysteine-rich motifs. The minimal part of PLSCR1 sufficient to mediate interaction with SLPI, and with the CD4 receptor (see below), was mapped within a 79 a.a. long region comprised between residues 155 and 233. This region of PLSCR1 contains the cysteine cluster that forms a site for palmitoylation involved in the stabilization of the membrane anchoring of PLSCR1 [28], and this cluster is conserved in PLSCR4. However, substitution of the 6 cysteine residues (Cys184 to Cys189) did not disturb the capacity of PLSCR1 to interact with either SLPI or CD4, indicating that the palmitoylation motif does not directly participate in these interactions. These cysteine residues may indirectly participate since palmitoylation is required for membrane expression of PLSCR1 which, when mutated, is found in the nucleus [32]. Similarly, we found that both PLSCR3 and PLSCR4 are primarily localized at the plasma membrane of T cells when they are expressed as GFP fusions. SLPI, which is secreted in the extracellular medium, therefore needs to cross the plasma membrane for efficient binding to the endofacial domain of PLSCR1 and PLSCR4. It has been recently documented that recombinant SLPI can be translocated through the plasma membrane to reach the cytoplasm and nucleus of cells such as monocytes, macrophages and B lymphocytes where it interacts with cellular effectors [3], [4], [33]; similarly, we confirmed that the purified GST-SLPI fusion protein used in the present study was also able to penetrate into the cells from the culture medium (data not shown).

While elafin is formed by a single WAP motif and interacted weakly with PLSCR1, SLPI contains two WAP motifs and each motif was able to support binding to PLSCR1, indicating that both domains may cooperate in an additive manner leading to an optimal interaction with PLSCR1. This also suggests that PLSCR1 binding is not mediated by a linear motif contained within the SLPI primary sequence, but is rather related to the recognition of the conformational structure of the WAP domains. Like other members of the WAP family proteins, SLPI is secreted by epithelial cells and is thus found in all mucosal fluids ([1], for review). However, more recent studies have revealed that SLPI can be also produced and secreted by cells of the immune system, including neutrophils, B lymphocytes, macrophages and dendritic cells [33]–[36]. This suggests that exogenous SLPI functions as more than a protease inhibitor of leukocyte serine proteases for the protection of tissues at sites of inflammation, but it also participates as an intracellular modulator in some signaling transduction pathways. Similarly, our observations suggest that SLPI is an intracellular ligand which may participate in the modulation of the PLSCR1 and PLSCR4 functions by interacting directly with their cytoplasmic tail.

The antiviral activity of SLPI against HIV-1 has been documented in several reports [7]–[11], [13]. SLPI is able to inhibit the replication of primary viral strains both in human primary CD4-positive T lymphocytes and in monocyte-derived macrophages at the physiological concentrations found in saliva (20–120 µg/ml). In addition, we show here that SLPI is also able to inhibit the capture of HIV-1 virions from dendritic cells derived from primary blood monocytes and then the transfer of virions towards autologous T lymphocytes, a process which is important *in vivo* for spreading of the virus after mucosal transmission (for review, [26]). This inhibitory activity is exerted on viral strains with tropism for either lymphocytes or macrophages. The inhibition of HIV-1 replication by SLPI is independent of its anti-protease activity, does not involve a direct action on virus particles, and is related to a block of the virus entry process at a stage that is posterior to the interaction of the viral gp120 envelope glycoprotein with the CD4 receptor [9], [11]. This antiviral activity is rather due to an interaction with the host cell surface molecule, and annexin II, a membrane-associated protein that is specifically expressed in macrophages, has been recently proposed as a receptor for SLPI that could participate in HIV-1 infection of macrophages [9]. However, this finding does not explain the ability of SLPI to block HIV-1 replication both in CD4-positive transformed T-cell lines and in primary peripheral blood lymphocytes [7], [8], [10], [13], [19] where annexin II does not seem to be expressed [11], [20]. In addition, it was recently reported that annexin II was rather involved in the late stage of HIV-1 assembly through a direct interaction with the viral Pr55Gag precursor on endosomal membranes of macrophages [20]. In this context, our findings raise a new hypothesis concerning the molecular mechanism of antiviral activity of SLPI. Our data suggest that SLPI may disrupt the interaction of CD4, the main receptor of HIV-1, at least with the PLSCR1 membrane protein at the cell surface of CD4-expressing cells. Although the functions of the interactions of CD4 with PLSCR1 and PLSCR4 in the biology of lymphoid or myeloid cells expressing CD4, as well as in the HIV-1 infection process, remain to be elucidated, the demonstration that the CD4 receptor associates directly with PLSCR1 and PLSCR4 may represent a critical point for the understanding of the antiviral activity of SLPI. While we cannot exclude that SLPI could act as a modulator on some other cellular signaling pathways leading to inhibition of HIV-1 replication [3], [4], [9], we propose that the antiviral activity of SLPI is related, at least in part, to the disruption of the interactions of CD4 with PLSCR1 and PLSCR4. As mentioned above, it is now clearly established that SLPI is indeed capable to cross the biological membranes to access the cytoplasm of the cell ([3], [4], and data not shown) where it could disrupt the interaction between the cytoplasmic domains of CD4 and PLSCR1 and PLSCR4.

In summary, the characterization of a direct interaction between CD4 and scramblases, proteins regulating the movement of phospholipids in the plasma membrane, as well as our finding that SLPI, an antiviral polypeptide, disrupts the interaction between CD4 and PLSCR1, open new directions for therapeutic interventions. We hypothesize that the data generated herein will help to the development of new antiviral strategies, and we are currently looking to identify chemical compounds disrupting the interaction between CD4 and PLSCR1 by conducting a high throughput screen using the ELISA test developed in the present study (see Fig. 6) as a primary interaction assay that recapitulates *in vitro* the association of CD4 with scramblases.

## Materials and Methods

### Plasmids

#### Yeast expression vectors

The vector for expression of the cytoplasmic domain of CD4 fused to LexA has been described [37]. The DNA sequence coding for PLSCR1 was amplified by PCR from a cDNA library [38] and cloned into the pGAD3S2X plasmid (Clontech) to generate the pGAD-PLSCR1 vector for expression of PLSCR1 fused to the Gal4 activation domain (Gal4AD). The deleted forms of PLSCR1 (see on Fig. 7) were amplified by PCR using pGAD-PLSCR1 as a template and appropriate primers. The PCR products were then inserted into the *Eco*RI-*Not*I sites of pGAD3S2X for expression of the Gal4AD fusions. The DNA sequence coding for the truncated form (aa 21–132) of the human SLPI deleted of the signal peptide sequence was synthesized in vitro by PCR using a set of specific primers, and then cloned into the *Eco*RI-*Bam*HI sites of the pLex10 plasmid (Clontech) to generate the pLex-SLPI vector for expression of the LexA-SLPI hybrid.

#### Bacterial expression vectors

The vectors for expression of the full length PLSCR1 and the truncated form fused to the glutathione-S-tranferase (GST) were made by subcloning restriction fragments from the pGAD-PLSCR1 and pLex-SLPI into the *Bam*HI-*Xho*I and *Eco*RI-*Xho*I sites of the plasmids pGEX4T-2 and pGEX4T-1 (Invitrogen), respectively. For expression of the GST-SLPI W1 and GST-SLPI W2 fusions, PCR fragments obtained using pLex-SLPI as a template and appropriate primers were inserted into the *Eco*RI-*Xho*I sites of the pGEX4T-1 plasmid. The vector for expression of GST-elafin (a.a. 61 to 117) was obtained by PCR amplification of the DNA coding sequence of trappin-2 (pre-elafin) with specific primers and using the pGE-SKAP plasmid (gift of Thierry Moreau, Tours, France) as a template, and the PCR product was inserted into pGEX4T-1 as indicated above.

#### Mammalian expression vectors

The vectors for expression of the wild type and deleted forms of CD4 have been described previously [37]. The vector for expression of the hemaglutinin(HA)-tagged form of PLSCR1 was made by PCR amplification using pGAD-PLSCR1 as a template and appropriate primers, and the PCR product was subcloned in the *Bam*HI-*Xho*I sites of the pAS1B plasmid [39]. Vectors for expression of the full length and truncated forms (1–310 and 1–290, see on Fig. 3) of PLSCR1 fused to GFP (GFP-PLSCR1) were made by PCR amplification using pGAD-PLSCR1 as a template; the PCR products were inserted into the *Xho*I-*Eco*RI sites of the pEGFPC1 plasmid (Clontech). Vectors for expression of GFP-PLSCR3 and GFP-PLSCR4 were made by PCR amplification using pENTR221-PLSCR3 and pENTR221-PLSCR4, purchased from Invitrogen (clones ID IOH13045 and IOH55049, respectively), as templates; the PCR products were then inserted as previously in pEGFPC1 (Clontech).

### Commercial antibodies and reagents

The monoclonal antibody used to immunoprecipitate CD4, OKT4 (ATCC), is specific for the extracellular domain of CD4. In western blot analysis, CD4 was detected using rabbit anti-CD4 polyclonal antibody H370 (Santa Cruz), while GFP-PLSCR1, -PLSCR3 and -PLSCR4 fusions were detected using a pool of two mouse anti-GFP monoclonal antibodies (7.1 and 13.1, Roche). The mouse anti-GST antibody (G1160) used in ELISA was from Sigma. Secondary reagents were horseraddish peroxidase-coupled anti-mouse and anti-rabbit IgG (Sigma), both used at 1∶2,000 dilution. Immunofluorescence staining was performed with anti-CD4 (SK3, Becton Dickinson) followed by goat anti-mouse Alexa594-coupled antibody (Invitrogen). Non-tagged SLPI was purchased from R&D, while the CD4 long (a.a. 405–433) and short (a.a. 405–426) synthetic peptides (>90% pure) were purchased from NeoMPS (Strasbourg, France).

### Generation of anti-PLSCR1 antibodies

Monoclonal antibodies against human PLSCR1 were generated as follows: a 6 week-old BALB/c mouse was subcutaneously immunized with GST-PLSCR1 (10 µg) emulsified in 100 µl Freund's complete adjuvant. Immunizations were repeated at 7-day intervals in incomplete Freud's adjuvant (3 times) and in PBS one day before cell fusion. Lymph node cells were fused with Ag8.653 mouse myeloma cell line (1∶1 ratio) [40]. Hybridoma supernatants specificity was tested by ELISA comparing GST and GST-PLCR1 recombinant proteins. All positive specific clones were then tested by immunoprecipitation and immunofluorescence. One hybridoma (clone 314A10, IgG1) was obtained after subcloning by limited dilution. Polyclonal antibodies were obtained by immunizing rabbits with the PLSCR1 C-terminal peptide (encompassing the 306–318 a.a. sequence CESTGSQEQSSGVW) as described elsewhere [23].

### Cell culture and transfection

CD4-positive HPB-ALL and Jurkat T-cells (clone 20) were kindly provided by G. Bismuth (Institut Cochin, Paris, France) and C. Hivroz (Institut Curie, Paris, France), respectively. These T-cell lines were maintained in RPMI 1640 medium with Glutamax-1 (Invitrogen) supplemented with 10% fetal calf serum (FCS) and 100 U/ml penicillin/streptomycin. HPB-ALL and Jurkat cell lines were electroporated using Biorad electroporator (Gene pulser II) at 250 V and 950 µF in complete media supplemented with 40 mM NaCl. 293T cells were grown in Dulbecco modified Eagle's medium (DMEM) supplemented with 10% FCS, 100 IU/ml penicillin and 0.1 mg/ml streptomycin. 293T cells were transfected using the calcium phosphate precipitation technique. Peripheral blood mononuclear cells (PBMC) were isolated from blood of healthy donors by Ficoll (MSL) density gradient centrifugation. Monocytes were isolated from PBMC by plastic adherence, and immature dendritic cells were obtained from monocytes differenciated in the presence of GM-CSF and IL-4 (both at 10 ng/ml) for 6 days of culture as described previously [41]. Contamination of immature dendritic cells with CD3+ T lymphocytes was <1% as checked by flow cytometry (data not shown). Autologous lymphocytes, corresponding to the non adherent fraction after the adherence step, were cultured in RPMI, 10% FCS, 1% antibiotics supplemented with PHA (2.5 µg/ml) and IL-2 (10 ng/ml) for 48 h. Cells were then washed and cultured for additional 24 h in the presence of IL-2. All cell lines were maintained in a humidified atmosphere at 37°C with 5% CO2.

### GST pull-down assay

Wild-type or truncated GST-SLPI fusions, as well as GST-PLSCR1, GST-Arf1 and GST-elafin fusions were produced in BL21 *E. coli* and then purified on glutathione(GSH)-sepharose beads (GE Healthcare) as described previously [42]. The lysates from 1×10^7^ Jurkat cells or 5×10^7^ 293T cells previously transfected with vectors for expression of either GFP-PLSCR1, GFP-PLSCR3 or GFP-PLSCR4 were incubated overnight at 4°C in the lysis buffer (Tris-HCl pH 7.6 20 mM, NaCl 140 mM, EDTA 1 mM, NP-40 1%, and antiprotease mixture from Sigma) with 2 µg of immobilized GST fusions. After 5 washes in the lysis buffer, bound proteins were separated by SDS-PAGE and analyzed by Western blotting using anti-CD4 H370 and anti-GFP antibodies. For inhibition experiments, GST-SLPI or GST-Arf1 (used as control) were initially eluted from beads with a GSH 50 mM, Tris 50 mM pH 8 buffer and dialyzed against PBS for 16 h at 4°C; lysates from Jurkat cells were then incubated with immobilized GST-PLSCR1 in the presence of 10 or 100 µg/ml of purified GST-SLPI, or in the presence of 120 µg/ml of purified GST-Arf1.

### Co-immunoprecipitation assay

For co-immunoprecipitation of endogenous CD4 and PLSCR1 proteins, Jurkat T cells were suspended in lysis buffer containing 100 mM (NH_4_)_2_SO_4_, 20 mM Tris (pH 7.5), 10% glycerol, 1% Igepal CA-630 and 1× complete protease inhibitor mixture (Roche Diagnostics GmbH). After 30-min incubation at 4°C under gentle agitation, cell lysates were centrifuged at 14,000 *g* for 30 min at 4°C. The soluble fraction was assayed for protein content with the DC protein assay kit (Bio-Rad). CD4 was immunoprecipitated from cleared lysates containing ∼2.5 mg of total proteins with 10 µg of OKT4, on protein A-Sepharose beads (Sigma-Aldrich). Either 20 or 80% of the precipitated material was resolved by SDS-PAGE and analyzed by Western blotting with an anti-CD4 (H370, Santa-Cruz, CA) and polyclonal antibodies generated against the C-terminal domain of PLSCR1, respectively.

The interactions between CD4 and either PLSCR1, PLSCR3 and PLSCR4 were also analyzed in 293T cells co-expressing CD4 and HA- or GFP-tagged forms of scramblases. CD4 was immunoprecipitated from cleared lysates containing ∼400 µg of total proteins with 2 µg of OKT4, on protein A-Sepharose beads and analyzed by Western blotting with anti-CD4 and either anti-HA or anti-GFP antibodies.

### ELISA interaction test

Microtiter plates (Fisher Scientific) were coated for 2 h at 37°C with the long or short CD4c synthetic peptides or with non-tagged SLPI at a final concentration of 1 µM and 0.25 µM in PBS, respectively. The wells were then washed 3 times in PBS/0.1% Tween 20 (PBS-T), and blocked for 16 h at 4°C with PBS-Tween supplemented with 0.1% bovine serum albumin (PBS-TB). After washings, the GST-PLSCR1 or GST-SLPI fusions were incubated for 2 h at 37°C at concentrations ranging from 1 nM to 1 µM in PBS-TB. The wells were then washed as previously, and an anti-GST was added to each well at a final concentration of 100 nM in PBS-TB. After 1 h at room temperature, the wells were washed again and incubated for 45 min at room temperature with a peroxidase-conjugated anti-mouse IgG Ab at a 1∶2,000 dilution in PBS-TB. The wells were washed and the TMB peroxidase substrate solution (BioRad) was added for a 10-min incubation at room temperature. The reaction was stopped by adding 50 µl of 2 M HCl per well. The optical density was measured at 450 nm with a VICTOR-V spectrophotometer from Perkin Elmer.

### Analysis of SLPI antiviral activity

For inhibition of HIV-1 infection of primary lymphocytes, lymphocytes were pre-incubated with purified GST-SLPI (6.25, 12.5, 25 and 50 µg/ml) for 30 min at room temperature before addition of HIV-1JR-CSF (1 ng/ml p24) for 1 h at 37°C. Cells were then extensively washed and cultured for 6 days. Supernatant was then collected and virus lysed with 0.5% Triton X-100 before determination of p24 levels by ELISA (Ingen). For inhibition of HIV-1 transmission in trans from dendritic cells to T lymphocytes, dendritic cells (1×10^5^ cells) were incubated with 1 ng/ml of either HIV-1Bal or HIV-1NDK p24 for 1 h at 37°C. Cells were extensively washed and IL-2-activated lymphocytes (5×10^5^ cells) were added and maintained in coculture in RPMI, 10% FCS, 1% antibiotics and supplemented with IL-2 (10 ng/ml) for 72 h. Supernatants were collected and HIV p24 levels in culture medium measured by ELISA. In inhibition experiments, cells were pre-incubated with purified GST-SLPI (50 µg/ml) for 30 min at room temperature before HIV-1 addition.

### Yeast two-hybrid system

All the procedures used to analyze interactions in the two-hybrid system were performed in the L40 yeast reporter strain as previously described in detail [37], [42].

### Immunofluorescence staining

24 h after transfection, HPB-ALL T cells (2×10^6^) were concentrated in 300 µl of culture medium without FCS and put on cover glass pretreated with poly-L-lysine (Sigma). After adhesion, cells were washed in PBS, fixed with 4% paraformaldehyde, and treated with 0.1 M glycine. Cells were then permeabilized with 0.1% Triton X-100 for 10 min. CD4 was detected with FITC-conjugated anti-CD4 (SK3, Becton Dickinson). Cells were then mounted in PBS containing 50% glycerol. Images were acquired with a Zeiss Axiophot microscope 80× equipped with a CCD camera controlled by Metaview software (Universal Imaging).
