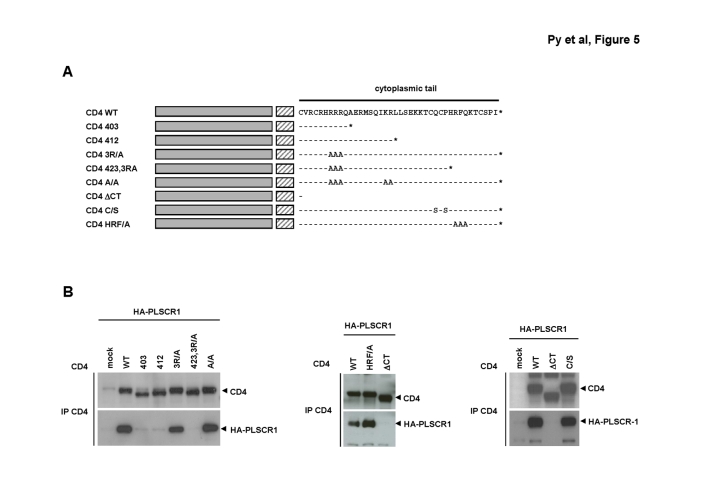# Correction: The Phospholipid Scramblases 1 and 4 Are Cellular Receptors for the Secretory Leukocyte Protease Inhibitor and Interact with CD4 at the Plasma Membrane

**DOI:** 10.1371/annotation/657cd713-aaac-4ebb-80ad-3ec8dfb12b42

**Published:** 2009-04-29

**Authors:** Bénédicte Py, Stéphane Basmaciogullari, Jérôme Bouchet, Marion Zarka, Ivan C. Moura, Marc Benhamou, Renato C. Monteiro, Hakim Hocini, Ricardo Madrid, Serge Benichou

The published Figures 2, 3, 4, and 5 are illegible. Please see the corrected figures here: 

Figure 2, 

**Figure:**
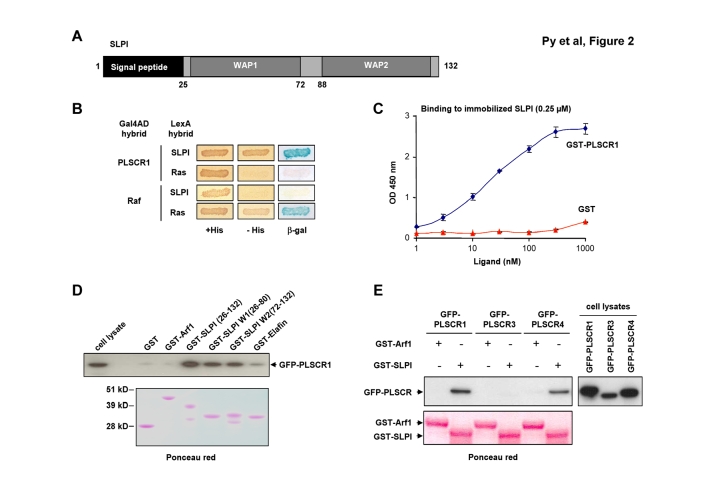


 Figure 3, 

**Figure:**
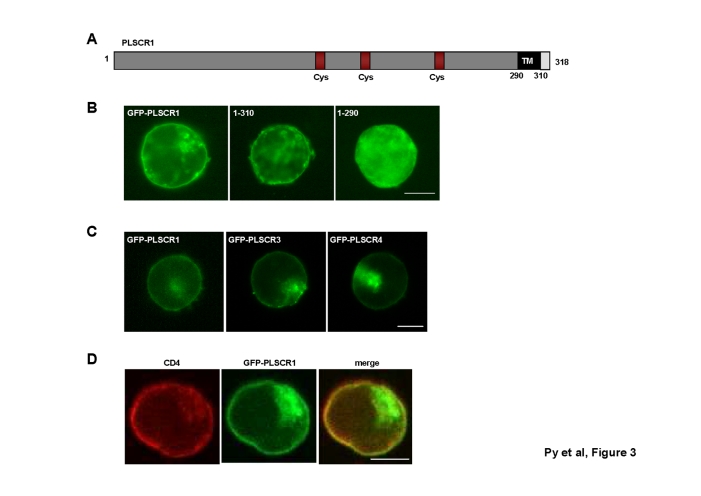


Figure 4, 

**Figure:**
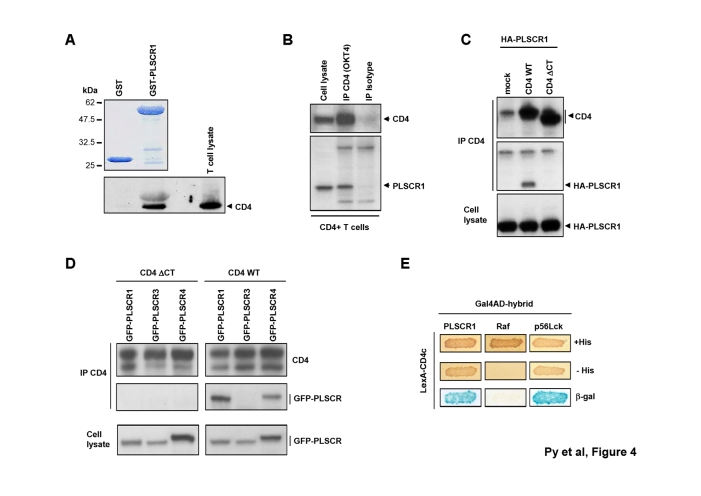


Figure 5, 

**Figure:**